# Validity of self-reported mammography uptake in the Belgian health interview survey: selection and reporting bias

**DOI:** 10.1093/eurpub/ckaa217

**Published:** 2020-11-23

**Authors:** Finaba Berete, Johan Van der Heyden, Stefaan Demarest, Rana Charafeddine, Jean Tafforeau, Herman Van Oyen, Olivier Bruyère, Françoise Renard

**Affiliations:** 1 Department Epidemiology and Public Health , Sciensano, Brussels, Belgium; 2 Department of Public Health, Epidemiology and Health Economics, University of Liège, Liège, Belgium; 3 Department of Public Health and Primary Care, Ghent University, Ghent, Belgium; 4 Department of Public Health, Epidemiology and Health Economics, WHO Collaborating Centre for Public Health Aspects of Musculoskeletal Health and Ageing, University of Liege, Liège, Belgium

## Abstract

**Background:**

The validity of self-reported mammography uptake is often questioned. We assessed the related selection and reporting biases among women aged 50–69 years in the Belgian Health Interview Survey (BHIS) using reimbursement data for mammography stemming from the Belgian Compulsory Health Insurance organizations (BCHI).

**Methods:**

Individual BHIS 2013 data (*n* = 1040) were linked to BCHI data 2010–13 (BHIS–BCHI sample). Being reimbursed for mammography within the last 2-years was used as the gold standard. Selection bias was assessed by comparing BHIS estimates reimbursement rates in BHIS–BCHI with similar estimates from the Echantillon Permanent/Permanente Steekproef (EPS), a random sample of BCHI data, while reporting bias was investigated by comparing self-reported versus reimbursement information in the BHIS–BCHI. Reporting bias was further explored through measures of agreement and logistic regression.

**Results:**

Mammography uptake rates based on self-reported information and reimbursement from the BHIS–BCHI were 75.5% and 69.8%, respectively. In the EPS, it was 64.1%. The validity is significantly affected by both selection bias {relative size = 8.93% [95% confidence interval (CI): 3.21–14.64]} and reporting bias [relative size = 8.22% (95% CI: 0.76–15.68)]. Sensitivity was excellent (93.7%), while the specificity was fair (66.4%). The agreement was moderate (kappa = 0.63). Women born in non-EU countries (OR = 2.81, 95% CI: 1.54–5.13), with high household income (OR = 1.27, 95% CI: 1.02–1.60) and those reporting poor perceived health (OR = 1.41, 95% CI: 1.14–1.73) were more likely to inaccurately report their mammography uptake.

**Conclusions:**

The validity of self-reported mammography uptake in women aged 50–69 years is affected by both selection and reporting bias. Both administrative and survey data are complementary when assessing mammography uptake.

## Introduction

Breast cancer is the most common cancer in terms of incidence among women both in developed and developing countries^1–3^ and the second cause of cancer death among women after lung cancer in most developed countries.^4^

Early detection of breast cancer through mammography screening is recognized as being effective in reducing mortality^5^^,^^6^; in women aged 50–69 years.^7–9^ Literature suggests that with a screening attendance reaching 70%, a reduction in breast cancer mortality by about 25% might be expected.^8^^,^^10^ European guidelines recommend biennial mammography screening for women aged 50–69 years.^11^

Valid methods of determining and monitoring breast cancer screening (screening) uptake are important to evaluate screening programs.^6^^,^^12^^,^^13^ Underestimating screening prevalence could lead to waste of resources, while overestimation could lead to missed opportunities for improving screening.^6^

Currently, information on screening is often based on self-reports in population-based surveys.^5^^,^^6^^,^^12^^,^^13^ Such information is used to monitor screening rates over time and to target interventions. However, the validity of self-reported information through surveys is a concern due to a potential selection (because of non-coverage or non-response error) and reporting bias associated with differential survey participation. Survey participants may systematically differ from the general population and reporting may be inaccurate due to memory and social desirability effects. The validity can also be different for different subpopulations. For example, it has been shown that members of ethnic minority groups and people with a lower socioeconomic status are more likely to inaccurately report cancer screening than their counterparts.^5^^,^^13^^,^^14^

According to the European screening quality assurance guidelines, the acceptable and desirable participation rates of screening are 70% and 75%, respectively.^9^ Furthermore, the European Partnership for Action Against Cancer called for reducing the burden of cancer by achieving 100% population coverage of screening for breast, cervical and colorectal cancer in 2013.^15^^,^^16^ In the USA, the Healthy People 2020 goals calls for a rate of adherence to national cancer-screening guidelines of 81% biannual mammography among women aged 50–74 years.^17^^,^^18^

To verify whether these goals are met, it is necessary to ensure that the data for estimating the national mammography uptake rate are valid.

The validity of self-reported mammography uptake can be verified by comparing this information with a trusted measure (gold standard). Numerous validation studies and meta-analyses have documented the level of agreement/disagreement between self-reported cancer screening and cancer registers, claims databases, electronic medical records and administrative data.^5^^,^^6^^,^^12^^,^^13^ They have reported a sensitivity between 95% and 97%, a specificity between 61% and 64% and have concluded that the estimates based on self-report are usually over-estimated.^5^^,^^12^ However, most of these validation studies are either limited to a specific geographical region^6^^,^^19–21^ or a specific subgroup^13^^,^^14^, leading to a problem of generalizability.

In Belgium, breast cancer is the first female cancer in terms of incidence (more than a third of cancers)^22^ and the leading cause of premature death among women.^23^

A national mammography screening program exists in Belgium since 2001–02. Mammograms realized within this organized screening program are called ‘mammotests’. Such mammograms are entirely reimbursed by the National Institute of Health and Disability Insurance (NIHDI) as well as diagnostic mammograms (i.e. among symptomatic women or those at high-risk). The mammotests and diagnostic mammograms are coded differently in the BCHI database. Besides the mammotests, a number of screening was often realized by women outside of the official screening program (by their own initiative). These later are called ‘opportunistic screening’ and are not reimbursed by the NIHDI. More often, for the reimbursement purposes, the opportunistic screening is miscoded as diagnostic mammograms. The proportion of mammograms realized outside of the screening program is important. Thus, information on mammography uptake gathered through the BCHI data allow to capture the total coverage of the screening than those through the official screening program. Each woman aged 50–69 years receives every 2 years an invitation to participate in the screening program. The mammograms realized within the program follow a specific procedure. The examination is free of charge.^24^ Exhaustive information on the mammography uptake is available through the Belgian Compulsory Health Insurance (BCHI) including both mammograms realized within and outside the organized screening program.^25^ However, the BCHI database is limited in terms of socio-demographic information.

Information on mammography uptake (‘having had mammograms’), based on self-reports is available in the Belgian Health Interview Survey (BHIS).^26^ The added value of the BHIS data is that it provides a comprehensive information on socioeconomic status (SES) and many other health-related topics useful for subgroup analyzes. Nevertheless, as in other population surveys, selection and reporting bias are also a concern in the BHIS.^27^ In this study, we investigate the validity of self-reported mammography attendance in the BHIS, as a proxy of screening uptake by assessing the associated selection and reporting biases.

## Methods

### Data sources

BHIS 2013 data were linked to BCHI 2010–2013 data (BHIS–BCHI) by means of a unique identifier (the national register number). The BHIS is a national, cross-sectional household survey conducted every 5 years since 1997 by Sciensano among a representative sample of Belgian residents. Participants are selected from the national population register through a multistage stratified sampling procedure. The detailed methodology of the survey is described elsewhere.^28^

The BHIS collects information on mammography uptake by means of a self-administered questionnaire in women aged 15 years and older (the reference population of Eurostat, although the main indicator refers to women aged 50–69 years): Have you ever had mammograms? ‘Yes/No’ and for those who respond ‘Yes’, the time lapse since her last mammograms: ‘When was the last time you had mammograms?’ Furthermore, the BHIS also collects data on a wide range of other health and health-related topics such as demographic information, SES and self-reported health status, life style and health services use. The BHIS has been approved by the ethics committee of the University hospital of Ghent on 1 October 2012 (advice EC UZG 2012/658). For the linkage, an authorization was obtained from the Belgian Privacy Commission.

BCHI data contain exhaustive and detailed information on the reimbursed health expenses of over 99% of the total population. The database also includes a limited amount of socio-demographic information.^29^

The Echantillon Permanent/Permanente Steekproef (EPS) data, representing 1/40 of the Belgian population, is an unbiased random sample of the BCHI and contains the same information as the BCHI (reimbursed medical acts, hospitalization and medicines) which is also followed over time. The use the data of this population cohort in an anonymized way for policy and research purposes is regulated by a specific legal framework.^27^ All women aged 50–69 years within the EPS are included in this study. The analysis of the EPS data does not require any design settings.

### Inclusion criteria

This study included women aged 50–69 years who responded to the questions related to the mammography uptake ‘having had mammograms’ of BHIS (*n* = 1081). Linkage with BCHI was possible for 1040 women (96%). To assess the validity of self-reported mammography uptake, reimbursement for a mammography within the last 2 years preceding the BHIS was used as the gold standard. As nor in BHIS nor in BCHI it is not possible to disentangle mammograms realized within the screening program from opportunistic screening, both types are included in this study. We assume that in both sources, the mammography uptake in this age-group is a good proxy for the screening uptake.

### Analyses

Mammography uptake rates by data source were calculated.

#### Selection bias

The potential selection bias was computed as the difference between the prevalence of register within BCHI based mammography uptake from the BHIS–BCHI and similar estimates from the EPS data (absolute bias), and dividing that difference by the prevalence from the EPS data and multiplied by 100 (relative bias).^30^ The 95% CI of the estimated bias was computed using the Delta method.^29^ Analyses were done overall and by age-group and region of residence.

#### Reporting bias

The reporting bias was assessed as the difference in the prevalence of mammography uptake between BHIS and BCHI estimates from the BHIS–BCHI linked data. As for the selection bias, both absolute and relative percentages were calculated. Next, the report-to-record ratio (RRR) was computed. The RRR is the ratio of the percentage of women reporting having had mammograms to the percentage of women reimbursed for mammograms during the relevant time period, and its confidence intervals. The RRR is frequently used as a measure of net bias of self-report, with values greater than one indicating over-reporting and values less than one indicating under-reporting.^6^^,^^13^^,^^31^^,^^32^ Furthermore, the sensitivity (i.e. the percentage of women classified as screened in the BHIS among those who were reimbursed for mammograms in the BCHI), the specificity (i.e. the percentage of women classified as not screened in the BHIS, among those who were not reimbursed for mammograms in the BCHI), the positive predictive value (PPV, i.e. the percentage of women reimbursed for mammograms in the BCHI, among those classified as screened in the BHIS) and the negative predictive value (NPV, i.e. the percentage of women who were not reimbursed for mammograms in the BCHI, among those who were classified as not screened in the BHIS) were calculated. These estimates were classified as excellent (>0.90), good (>0.80), fair (>0.70) or poor (<0.70).^33^ Sensitivity analysis was performed by moving the time frame for screening from 2 to 3 years. The total agreement as well as the Cohen’s kappa statistic were also calculated to provide a measure of agreement beyond chance.^34^ Cutoffs used to classify kappa are based on McHugh et al.: 0–0.20 = none agreement; 0.21–0.39 = minimal agreement; 0.40–0.59 = weak agreement; 0.60–0.79 = moderate agreement; 0.80–0.90 = strong agreement; above 0.90 = almost perfect agreement.^35^

The calculations were done for the whole population and by socio-demographic subgroups; by age-group (50–59 years, 60–69 years), educational level, country of birth (Belgium, other EU country, non-EU country), region of residence (Flanders, Brussels and Wallonia), income category (low, high) and self-perceived health (good to very good, very bad to fair). Educational level was based on the highest level of education achieved in the household according to the ISCED 1997^36^ and recoded into three categories: low (lower secondary education or less), intermediate (higher secondary education) and high (higher education). For income level, the quintiles of the equivalent household income were recoded in low (quintile 1–3) and high (quintile 4 and 5). As this is an exploratory and post-hoc analysis of existing data, a strict adjustment for multiple comparisons is less critical.^37^ Therefore, we declined to adjust for multiple comparisons.

Finally, multivariable logistic regression was used to identify covariates associated with inaccurate self-reported mammography uptake (over- or under-reporting). All variables cited above were included as independent variables. In order to maximize the information available in the analyses and to prevent potential bias caused by selective drop out, item non-response for education, household income, perceived health and place of birth (item missingness between 1% and 10%) was addressed by multiple imputations. Age, region of residence, as well as the dependent variable were used in the imputation model. The dependent variable (3.5% of missingness) was included in the imputation model in order to enhance it and was reliably imputed. However, its imputed values were not used in the analysis model. Multivariate normal regression was used as the imputation method to estimate missing values.^38^ Survey data were analyzed taking into account the multistage stratified clustered sampling design of the BHIS: use of post stratification weights, geographical stratification at the level of the province and clustering at household level. Statistical significance was defined as *P* < 0.05. Potential selection and reporting bias were estimated using Stata 15.1©. All the remaining analyses were performed using SAS 9.4^©^.

## Results

Table 1 presents the prevalence of mammography uptake by data source and subgroups. Based on the BHIS–BCHI, the mammography uptake in the BHIS 2013 sample was estimated to be 75.5% using BHIS information and 69.8% using BCHI information. Within the EPS, the percentage was 64.1%. The percentage also varies significantly across subgroups in both data sources.

**Table 1:** Prevalence of mammography uptake in the last 2 years, by source and subgroups, Belgium 2013

	BHIS–BCHI linked (*n* = 1040)				EPS (*n* = 36 700)	
	BHIS		BCHI			
	% uptake	95% CI	% uptake	95% CI	% uptake	95% CI
Overall	75.5	(72.1–78.9)	69.8	(66.2–73.4)	64.1	(63.6–64.6)
Age (years)						
50–59	78.0	(73.6–82.4)	68.6	(63.6–73.6)	67.1	(66.4–67.7)
60–69	72.8	(67.6–77.9)	71.1	(65.8–76.3)	67.0	(66.3–67.8)
Educational level					NA	
Low	66.2	(59.4–73.1)	61.0	(53.5–68.4)		
Middle	76.4	(70.3–82.4)	73.4	(67.1–79.6)		
High	81.7	(76.9–86.4)	73.7	(68.2–79.2)		
Place of birth					NA	
Belgium	76.0	(72.4–79.5)	70.1	(66.3–73.9)		
EU country	63.2	(50.1–76.3)	57.5	(44.0–71.1)		
Non-EU country	82.7	(66.8–98.6)	81.0	(65.0–97.0)		
Region						
Flanders	78.0	(73.4–82.6)	76.1	(71.2–80.9)	71.2	(70.6–71.8)
Brussels	75.8	(68.4–83.2)	66.7	(58.5–74.9)	59.3	(57.40–61.2)
Wallonia	70.3	(64.9–75.6)	57.3	(51.4–63.2)	61.8	(61.0–62.8)
Income					NA	
Low	71.9	(66.6–77.1)	66.2	(60.7–71.6)		
High	79.4	(74.5–84.3)	74.6	(69.2–80.0)		
Health status					NA	
Good to very good	79.7	(75.8–83.6)	74.0	(69.7–78.2)		
Very bad to fair	65.0	(58.6–71.3)	58.6	(51.8–65.4)		

BHIS, Belgian Health Interview Survey; BCHI, Belgian Compulsory Health Insurance; EPS, Permanent Sample (random sample of the Belgian Compulsory Health Insurance data); NA, Not available.

Table 2 summarizes both the selection and reporting biases. A significant difference between the BHIS–BCHI and the EPS mammography uptake reimbursement rates is observed overall. The absolute and relative size of the selection bias is 5.72% points (95% CI: 2.06–9.38) and 8.93% (95% CI: 3.21–14.64), respectively. No significant differences were detected between subgroups.

**Table 2:** Estimated bias in mammography uptake in the last 2 years among women aged 50–69 years in the BHIS–BCHI linked sample, Belgium 2013

	**Estimated bias** ^a^				
	**Selection bias** ^b^ **(%)**		**Reporting bias** ^c^ **(%)**		
	**Absolute** ^d^ **(95% CI)**	Relativee (95% CI)	**Absolute** ^f^ **(95% CI)**	**Relative** ^g^ **(95% CI)**	RRR (95% CI)
Overall	5.72 (2.06–9.38)*	8.93 (3.21–14.64)*	5.74 (0.75–10.72)*	8.22 (0.76–15.68)*	1.08 (1.02–1.15)*
Age (years)					
50–59	1.56 (−3.50–6.62)	2.32 (−5.23–9.87)	9.42 (2.67–16.17)*	13.72 (3.12–24.33)*	1.14 (1.05–1.23)*
60–69	4.04 (−1.27–9.34)	6.02 (−1.91–13.95)	1.67 (−0.57–9.06)	2.35 (−8.16–12.87)	1.02 (0.94–1.11)
Educational level	N.A^h^				
Low			5.22 (−5.09–15.53)	8.56 (−9.11–26.23)	1.09 (0.95–1.24)
Middle			2.99 (−5.67–11.65)	4.08 (−7.97–16.12)	1.04 (0.95–1.14)
High			7.98 (0.62–15.34)*	10.83 (0.22–21.45)*	1.11 (1.02–1.21)*
Place of birth	N.A^h^				
Belgium			5.86 (0.63–11.10)*	8.36 (0.56–16.17)*	1.08 (1.02–1.15)*
EU country			5.67 (−14.66–26.01)	9.86 (−27.25–46.97)	1.10 (0.90–1.34)
Non-EU country			1.70 (−19.67–23.07)	2.10 (−24.54–28.74)	1.02 (0.83–1.25)
Region	N.A^h^				
Flanders	4.86 (−0.03–9.75)	6.82 (−0.06–13.70)	1.95 (−4.75–8.66)	2.57 (−6.36–11.50)	1.03 (0.95–1.11)
Brussels	7.43 (−0.89–15.75)	12.53 (−1.60–26.67)	9.12 (−1.83–20.01)	13.67 (−4.00–31.34)	1.14 (1.00–1.29)
Wallonia	−4.56 (−10.51–1.38)	−7.38 (−16.98–2.22)	12.95 (5.01–20.89)*	22.60 (6.95–38.25)*	1.23 (1.10–1.36)*
Income	N.A^h^				
Low			5.69 (−1.86–13.25)	8.61 (−3.33–20.54)	1.09 (0.99–1.19)
High			4.82 (−2.54–12.19)	6.46 (−3.76–16.69)	1.05 (0.97–1.13)
Health status	N.A^h^				
Good to very good			5.69 (−0.07–11.44)	7.69 (−0.41–15.79)	1.08 (1.01–1.15)
Very bad to fair			6.41 (−3.15–15.98)	10.96 (−6.32–28.23)	1.11 (0.98–1.26)

a Computed before rounding the percentages.

b Computed by comparing the percentage of women with a mammography reimbursement in the BHIS–BCHI linked sample and in the EPS data.

c Computed by comparing the percentage of self-reported mammography uptake and mammography reimbursement in the BHIS–BCHI linked data.

d Absolute difference in the prevalence of mammography reimbursement rates in the BHIS–BCHI linked sample and similar estimates from the EPS data.

e Relative excess in percentage, computed as the differences between the percentage of women with a mammography reimbursement in the BHIS–BCHI linked sample and in the EPS data, divided by the percentage from the EPS data.

f Absolute difference in the prevalence of self-reported mammography uptake and reimbursement rate in the BHIS–BCHI linked data.

g Relative excess in percentage, computed as the difference between the percentage of self-reported mammography uptake and mammography reimbursement in the BHIS–BCHI linked data, divided by the percentage of reimbursement in the BHIS–BCHI linked data.

h N.A = Not available.

* Significant result (*P* < 0.05).

Also, for the reporting bias, a significant difference between self-reported and reimbursement information in the BHIS–BCHI is observed. The absolute size is 5.74% points (95% CI: 0.75–10.7) and the relative size is 8.22% (95% CI: 0.76–15.68), respectively. A subgroup analyses indicates that the mammography uptake is over reported by 14% for women aged 50–59 years, 11% for those highly educated, 8% for women born in Belgium and 23% for those residing in Wallonia. This over-reporting is confirmed by the RRR in the related subgroups.

Table 3 reports the more common measures of agreement related to the reporting bias. The sensitivity was excellent overall (93.7%) and across subgroups except for women born in other EU countries and for those reporting a poor perceived health; whereas the specificity was poor (66.4%) overall and did not exceed 70% in most of the subgroups. When the time frame was moved from 2 to 3 years, the specificity increased to 83%. The overall agreement was 84% (result not shown) and the kappa statistics was 0.63. The PPV was good overall and in all subgroups except for women born in non-EU countries where it was excellent. The NPV was above 80% in all subgroups but fair for women aged 60–69 years and those with middle educational level.

**Table 3:** Measures of validity of self-reported mammography uptake using administrative data as gold standard (BHIS–BCHI linked), Belgium 2013

Characteristics	Sensitivity (%)	Specificity (%)	Positive predictive value (%)	Negative predictive value (%)	Kappa statistic
Overall	93.7 (91.5–95.9)	66.4 (59.7–73.1)	86.6 (83.5–89.6)	82.0 (76.2–87.8)	0.63 (0.58–0.68)
Age (years)					
50–59	95.9 (93.1–98.6)	61.0 (51.5–70.4)	84.3 (79.9–88.7)	87.1 (78.9–95.3)	0.63 (0.56–0.70)
60–69	91.4 (87.8–94.9)	73.0 (64.0–81.9)	89.3 (85.3–93.3)	77.4 (68.9–85.9)	0.64 (0.56–0.71)
Education					
Low	98.4 (84.1–94.7)	70.0 (58.9–81.1)	82.3 (75.2–89.5)	80.8 (71.1–90.6)	0.60 (0.50–0.70)
Middle	92.1 (87.7–96.4)	66.9 (54.1–79.7)	88.5 (83.6–93.3)	75.4 (62.1–88.7)	0.59 (0.50–0.69)
High	97.5 (95.0–99.9)	62.6 (50.7–74.5)	87.9 (83.3–92.6)	89.8 (80.7–98.9)	0.70 (0.62–0.78)
Place of birth					
Belgium	93.8 (91.4–96.1)	65.7 (58.6–72.8)	86.5 (83.3–89.7)	81.8 (75.6–88.0)	0.64 (0.58–0.69)
EU countries	88.0 (78.1–97.8)	70.4 (50.3–90.5)	80.1 (66.0–94.2)	81.2 (64.6–97.9)	0.53 (0.34–0.72)
Non-EU country	99.0 (96.9–100)	86.7 (67.9–100)	96.9 (92.9–100)	95.2 (83.8–100)	0.77 (0.55–0.98)
Region					
Flanders	92.6 (89.6–95.7)	68.4 (57.5–79.3)	90.3 (86.5–94.1)	74.5 (64.7–84.2)	0.61 (0.52–0.70)
Brussels	98.5 (96.7–100)	69.6 (55.7–83.5)	86.7 (80.0–93.3)	95.9 (90.9–100)	0.72 (0.62–0.83)
Wallonia	95.5 (92.8–98.1)	63.6 (54.8–72.3)	77.9 (71.9–83.8)	91.3 (86.2–96.4)	0.60 (0.53–0.67)
Income category					
Low	93.7 (90.3–97.1)	70.9 (62.7–79.0)	86.3 (82.0–90.5)	85.2 (77.5–92.9)	0.64 (0.57–0.71)
High	93.6 (90.3–96.9)	62.2 (49.5–74.9)	87.9 (83.2–92.6)	76.7 (67.4–86.0)	0.61 (0.53–0070)
Health status					
Good to very good	95.2 (92.9–97.5)	64.6 (55.7–73.4)	88.4 (85.1–91.8)	82.6 (74.8–90.4)	0.65 (0.58–0.71)
Very bad to fair	89.1 (83.7–94.5)	69.1 (58.9–79.3)	80.3 (73.3–87.3)	81.8 (72.5–91.0)	0.60 (0.51–0.69)

The results of the multivariate logistic are shown in table 4. Inaccurate self-reported mammography uptake is more common among women born in a non-EU country (OR = 2.81, 95% CI: 1.54–5.13), people with a high household income (OR = 1.27, 95% CI: 1.02–1.60) and those reporting very bad to fair perceived health.

**Table 4:** Adjusted odds ratios (with 95% CI) of inaccurate self-reported mammography uptake in the past 2 years (defined as over-reporting or under-reporting). Results of multivariate logistic regression, Belgium 2013

Characteristics	OR (95% CI)
Age (years)	
50–59	1.00
60–69	1.05 (0.86–1.29)
Educational level	
Low	1.32 (0.97–1.80)
Middle	1.06 (0.79–1.41)
High	1.00
Place of birth	
Belgium	1.00
EU country	1.35 (0.77–2.35)
Non-EU country	2.81 (1.54–5.13)*
Region	
Flanders	1.00
Brussels	1.29 (0.92–1.82)
Wallonia	1.08 (0.83–1.41)
Income	
Low	1.00
High	1.27 (1.02–1.60)*
Health status	
Good to very good	1.00
Very bad to fair	1.41 (1.14–1.73)*

* Significant result (*P* < 0.05).

## Discussion

The main objective of this study was to assess the validity in terms of selection and reporting bias of self-reported mammography uptake in the BHIS. In the BHIS as in other interview surveys, the validity of self-reported information depends both on the selection and reporting bias. Our results indicate that the mammography uptake in the BHIS is significantly affected by both types of biases. Therefore, cautiousness is needed when using self-reported estimates as the sole method to quantify mammography coverage.

Due to the compulsory nature of the Belgian health insurance and the fact that the Belgian federal and regional governments signed a protocol agreement in 2001 for an organized screening program for women aged 50–69 years, to be organized by the regional government with appropriate financial resources supplied by the federal government, it can be stated that indicators based on the BCHI are quite reliable.

We found a significant selection bias. The relative overestimation of self-reported information was 9% overall.

Mammography uptake is also significantly affected by reporting bias in the same direction and in a comparable manner. Indeed, the relative overestimation of the percentage from the BHIS is 8% overall. This significant overestimation is observed across subgroups. Theoretically, the over-reporting could be partially due to an incomplete recording in the BCHI,^5^^,^^39^ but this is highly unlikely because for the financial management of the health insurance accurate data are essential. Therefore, administrative mistakes made by health insurance employees can be considered to be negligible. Another potential explanation is the underestimation of the timeframe since the last exam. This phenomenon, also called ‘telescoping’ (i.e. remembering that an event occurred more recently than it actually did), is the most consistent finding among studies comparing self-reports with medical or administrative data sources.^12^^,^^20^^,^^40^

The poor specificity found in our study (<70%) suggesting a higher rate of false positives could confirm the hypothesis of telescopic bias. We found that the telescopic bias represents almost half of the false positive cases. Indeed, if the time frame was moved from 2 to 3 years, the specificity would have been 83%. Over-reporting may also occur because adhesion to screening recommendations is perceived to be socially desirable.^12^ As opposed to findings in the literature,^6^^,^^13^ our results did not show that over-reporting mammography uptake occurred more often among women with a lower socioeconomic status. On the contrary, our results suggested that women with high household income level are more likely to inaccurately report (over-report) their mammography uptake.

When adjusted for other variables, women born in a non-EU country are more likely to inaccurate report (over-report) their mammography uptake as opposed of results from tables 2 and 3.

In the complete case analysis (results not shown), only the place of birth was significantly associated with inaccurate report of mammography uptake, probably because of loss of power due to drop out of missing values. Although the other variables were not significantly associated with the outcome, the direction of the effect remains unchanged as in analysis after multiple imputation.

Other validation studies have found results that are in line with those in our study. In their meta-analysis, Howard et al.^12^ estimated the pooled sensitivity and the pooled specificity to 95% and 62%, respectively. In another meta-analysis, Anderson et al. ^5^ also found excellent sensitivity (96%) but moderate specificity (61%). In another study, the specificity was much lower (45%) while the sensitivity was comparable.^40^ The authors explained this difference by the higher underestimation of the time elapsed since the last exam.

An important advantage of our study compared to most other studies is the fact that it was conducted in a representative sample of the population. The most common data used as gold standard in validation studies are medical records,^12^^,^^32^^,^^40^ which can be considered as more accurate than administrative data. However, medical data could be too difficult and expensive to obtain for population estimates. In our context, the use of administrative data as the gold standard is acceptable since they give exhaustive and accurate information on the number of mammograms that are carried out. Therefore, similar measures of validity (sensitivity, specificity) can be used as in studies that used medical records data as gold standard. The overall agreement (84.4%—result not shown) and the kappa statistic (0.63) as measures of reliability observed in our study were comparable to those in other studies.^32^^,^^40^

Another important strength of the current study is that we assessed concomitantly the selection and the reporting bias.

Some limitations of this study need to be highlighted. First, no distinction could be made between mammograms as part of a screening program and opportunistic mammograms in the BHIS. Moreover, because opportunistic screening mammograms are often miscoded as diagnostic mammograms for reimbursement purposes in the BCHI, we were unable to distinguish screening mammograms from diagnostic mammograms. However, since the proportion of diagnostic mammograms among all mammograms is quite low, the rate of mammograms outside the screening is an acceptable proxy of the opportunistic screening. So, the actual indicator that was assessed was ‘having had mammograms’, including both screening and opportunistic mammograms. The share of each type has never been measured in Belgium. In this study, we assumed that the largest part of the mammograms undergone between 50 and 69 is made for screening purposes, and therefore we used this information as a proxy of the breast cancer screening. Second, only a subpopulation of the BHIS participants (women aged 50–69 years) is analyzed. Ideally, a re-calibration of sample weights will be optimal. Unfortunately, because of the limited number of demographic variables in the reference dataset, this was not possible. Third, although it may seem more logical if we would have compared estimates obtained in the BHIS with screening information from the complete population, the data protection authority does not allow the use of exhaustive information from the BCHI if equally reliable information can be obtained from the EPS. As the EPS is a large sample and selected through a random procedure, it can be assumed that the EPS estimates perfectly match the indicators that would have been obtained from the total population.

This study has implications for public health policy-makers. Self-reported mammography uptake is not the most accurate method to track the national screening coverage rate and to determine the adherence to the national or international guidelines or attainment of goals. Therefore, the self-reported mammography uptake should be interpreted with caution and when possible objective data should be used.

Despite the moderate validity of mammography uptake in the BHIS, this data source still has an added value since it provides information on the socio-demographic determinants of the mammography attendance, and the link with health behaviors and other health outcomes.

## Conclusions

In the BHIS as in other interview surveys, the validity of self-reported information depends both on the selection and reporting bias. Our results indicate that the mammography uptake in the BHIS is significantly affected by both types of biases. Therefore, cautiousness is needed when using self-reported estimates as the sole method to quantify mammography coverage. Despite the moderate validity of mammography uptake in the BHIS, this data source still has an added value since it provides information on the socio-demographic determinants of the mammography attendance, and the link with health behaviors and other health outcomes. Further dedicated studies are needed to confirm our findings.
